# Assessment of the effects and limitations of the 1998 to 2008 Abbreviated Injury Scale map using a large population-based dataset

**DOI:** 10.1186/1757-7241-19-1

**Published:** 2011-01-07

**Authors:** Cameron S Palmer, Melanie Franklyn

**Affiliations:** 1 Trauma Service, The Royal Children's Hospital Melbourne, Flemington Rd, Parkville 3052, Australia; 2 Department of Mechanical Engineering, The University of Melbourne, 3010, Australia

## Abstract

**Background:**

Trauma systems should consistently monitor a given trauma population over a period of time. The Abbreviated Injury Scale (AIS) and derived scores such as the Injury Severity Score (ISS) are commonly used to quantify injury severities in trauma registries. To reflect contemporary trauma management and treatment, the most recent version of the AIS (AIS08) contains many codes which differ in severity from their equivalents in the earlier 1998 version (AIS98). Consequently, the adoption of AIS08 may impede comparisons between data coded using different AIS versions. It may also affect the number of patients classified as major trauma.

**Methods:**

The entire AIS98-coded injury dataset of a large population based trauma registry was retrieved and mapped to AIS08 using the currently available AIS98-AIS08 dictionary map. The percentage of codes which had increased or decreased in severity, or could not be mapped, was examined in conjunction with the effect of these changes to the calculated ISS. The potential for free text information accompanying AIS coding to improve the quality of AIS mapping was explored.

**Results:**

A total of 128280 AIS98-coded injuries were evaluated in 32134 patients, 15471 patients of whom were classified as major trauma. Although only 4.5% of dictionary codes decreased in severity from AIS98 to AIS08, this represented almost 13% of injuries in the registry. In 4.9% of patients, no injuries could be mapped. ISS was potentially unreliable in one-third of patients, as they had at least one AIS98 code which could not be mapped. Using AIS08, the number of patients classified as major trauma decreased by between 17.3% and 30.3%. Evaluation of free text descriptions for some injuries demonstrated the potential to improve mapping between AIS versions.

**Conclusions:**

Converting AIS98-coded data to AIS08 results in a significant decrease in the number of patients classified as major trauma. Many AIS98 codes are missing from the existing AIS map, and across a trauma population the AIS08 dataset estimates which it produces are of insufficient quality to be used in practice. However, it may be possible to improve AIS98 to AIS08 mapping to the point where it is useful to established registries.

## Background

Accurately determining the burden of significant injury within a given population is an essential role of a trauma system. Trauma data can be used for a range of applications including monitoring changes in injury epidemiology, trauma management and outcome over time, appropriate direction of funding, and population or outcome comparisons between institutions or regions. In order to monitor trauma systems most effectively, trauma registries should use contemporary terminology and tools; ideally, data collected using these tools should also be readily comparable.

The Abbreviated Injury Scale (AIS) [1] is widely used to monitor and evaluate anatomic injuries within trauma populations. Its utility has increased with the development of AIS-derived scores such as the Injury Severity Score (ISS) [2] and the New Injury Severity Score (NISS) [3]. The 1990 AIS version and its 1998 update (AIS98) [4] were extensively adopted [5,6] and large registries based on these coding systems exist. However, the epidemiological, diagnostic, management and outcome expectations inherent in these AIS versions (both in the available codes and in the severities assigned to each code) have been superseded by the 2005 AIS version (AIS05) [7] and its subsequent 2008 minor update (Abbreviated Injury Scale 2005 Update 2008 - AIS08) [8].

In order for trauma registries to reflect current management and treatment, the more contemporary AIS08 should be adopted. However, for established registries, there are significant limitations regarding the comparison of existing (AIS98) data to datasets compiled using AIS05 or AIS08 due to the magnitude of the changes made between the versions. The AIS codeset has expanded in size from 1341 codes in AIS98 to 1983 codes in AIS05, and then 1999 codes in AIS08. New sections allowing coding of drowning, asphyxia, and caustic corrosion injuries have also been included. Additionally, the consensus-derived severities of hundreds of codes have changed to reflect current clinical relevance in injury diagnosis, management and outcome [5]. For example, due to better treatment and management, many injuries will be assigned an AIS08 code which is lower in severity than its AIS98 equivalent.

Due to the differences between AIS versions, the creators of AIS developed a mapping system to convert data from AIS98 to AIS05/08. This mapping system (denoted 'dictionary map' from this point on) consists of two additional lists of AIS98 codes in the AIS05 and AIS08 dictionaries, which can be used to convert AIS98 data to AIS05/08 or vice versa. In theory, the dictionary map can be used to generate estimates of the data (for individual injuries, patients or an entire population) which would have been produced had the data been directly coded using the other version. For example, applying the dictionary map to AIS98-coded registry data should provide an AIS08-based injury dataset comparable to the dataset produced from directly coded AIS08 data. It has been asserted that by using the dictionary map, "conversions to and from AIS 98... should be relatively easy" [5].

A recent study conducted within the Victorian State Trauma System (VSTS) [9] demonstrated that the AIS05 dictionary map provided AIS estimates which gave a reasonable degree of comparability between mapped and double-coded data. In this study, mapping 'backwards' (i.e. from AIS05 to AIS98) gave slightly better performance than mapping 'forwards' (i.e. from AIS98 to AIS05), a result which might be expected as AIS05 contains considerably more codes and specificity than AIS98. However, although this study concluded that backwards mapping was preferable given the currently available mapping tools, there are significant objections to using backwards mapping in practice. Firstly, mapping AIS05/08 codes back to AIS98 will be impractical when successive AIS versions are introduced. Secondly, backwards mapping from the more contemporary AIS05/08 to AIS98 will result in assessments of patients' injuries which are outdated. Finally, it is simply illogical to adopt a contemporary and more complicated AIS version, only to convert the resultant data back to an outdated earlier version.

A number of recently published studies [9-12] identified differences between data coded using AIS98 and data coded using AIS05/08 in terms of the number of patients classified as 'major trauma' - an important, if often arbitrary discriminator between severely and less severely injured patients. Using the common threshold of an ISS >15 [13], the number of patients classified as major trauma has been estimated to decrease by between 8% and 24% using AIS05/08 when compared with AIS98. However, there are a number of potential limitations with these estimates. The smallest of these estimates was derived from a sample of patients with maxillofacial injury [12]. It has been noted that severity differences between AIS05/08 and earlier versions are not as great for facial injuries [9,14]. The three remaining studies were conducted in large (Level I) trauma centres. It is likely that the injury profile of trauma patients at these hospitals will differ from the injury profile of an entire trauma system. Finally, two of these studies [9,10] included only major trauma patients. It is possible that some less severely injured patients treated at these institutions sustained injuries which increased in severity using AIS05/08 (potentially increasing their ISS from below to above 15 in AIS05/08). Such patients would offset the noted decrease in major trauma numbers. Salottolo et al [11], who evaluated both major and non-major trauma patients, noted that approximately 1% of patients had an AIS05-based ISS which was in a higher ISS bracket than their AIS98-based ISS.

Additional problems have arisen due to the increased specificity in AIS05/08, where some injury types which were represented by only one code in AIS98 now have two or more corresponding codes in AIS08. A commonly occurring example would be a small (less than 1 cm thick), unilateral cerebral subdural haematoma (SDH). When directly coding this injury, it would be assigned an AIS severity level of 4 (severe) in AIS98; in AIS08, the injury can be assigned either level 3 (serious) or level 4, depending on if the thickness of the haematoma (in this instance, more or less than 6 mm thick). However, mapping of the AIS98 code to AIS08 would allocate the level 4 AIS08 code for all instances. This could potentially affect comparisons of, for example, ISS or NISS between patients whose data had been mapped from AIS98, and patients who were directly coded using AIS08.

In addition to AIS codes, many trauma registries and crash databases collect free text descriptions of each injury, documented by the injury coder at the time the AIS code is assigned and based on available information identified from the patient's medical file. Alongside a 'small cerebral SDH' AIS98 code, for example, a description of "5 mm right parietal SDH" may be recorded. Free text descriptions have been used in previous trauma research; in the landmark Major Trauma Outcome Study, the study method required that such descriptions were the sole source of information used to assign both AIS codes (in two separate versions) and International Statistical Classification of Disease (ICD) codes [15]. Where available, it is possible that free text information could be used to improve the accuracy of AIS mapping without needing to re-code patients from medical records. However, this concept has not previously been tested.

A final limitation of AIS mapping which has been identified is the occurrence of codes which have not been assigned equivalents in the dictionary map. In the VSTS double-coding study [9], 11% of patients could not have an ISS calculated from data which had been mapped from AIS98 to AIS05. This issue is therefore potentially significant, but has not been sufficiently explored. Examples from AIS98 are the codes for 'closed or undisplaced humerus fracture', or the combined code for 'fracture of 1 rib with haemo- or pneumothorax'. In many instances, it is unlikely that this has occurred due to an equivalent code not existing in AIS05/08, but rather in order to keep the dictionary map simple to read and use. In the first example, the AIS05/08 code for (non-open) humerus fracture (not further specified; NFS) has already been assigned an equivalent AIS98 code when mapping from AIS98 (that for 'humerus fracture NFS'). Codes for single rib fractures, haemothorax and pneumothorax also exist in AIS05/08, but a map between these and the combined code in AIS98 (where a patient has sustained two injuries) would be difficult to represent. Such issues are believed to affect forwards mapping from AIS98 more than backwards mapping from AIS05/08.

### Objectives

The aim of the current study was to determine how changes in injury severity between the AIS98 and AIS08 dictionaries might affect a large, established trauma registry when existing (AIS98-coded) data is mapped to AIS08 using the available dictionary map. More specifically, the effect of AIS version change on major trauma numbers will be ascertained by evaluating both major and non-major trauma patients. Additionally, the quality of data conversion from AIS98 to AIS08 will be assessed by examining the effect of injuries which cannot currently be mapped, or injuries which have more than one available mapping option. Finally, the potential for free text injury descriptions to improve data conversion from AIS98 to AIS08 will be examined by evaluating cerebral SDH injuries.

## Method

The Victorian State Trauma Registry (VSTR), a large, well-established population-based registry, was queried for the eight year period from 1st July 2001 to 30th June 2009 in order to identify all injuries sustained by patients meeting VSTR inclusion criteria. The VSTR collects data on patients with acute-phase injury who are transferred to or received from a VSTS hospital, or who have a length of stay greater than 72 hours [16]; consequently, patient data was not limited to major trauma. In addition to an ISS >15, patients could be defined as major trauma if they met any of the additional Victorian major trauma criteria - death due to injury, injury requiring urgent surgery, or an ICU stay of more than 24 hours with mechanical ventilation [16]. The information obtained included the VSTR patient number, the assigned AIS98 code (with corresponding free text description as written by VSTS coders) and the determined major trauma status (including which of the Victorian criteria were met).

The AIS98 to AIS08 dictionary map in the AIS08 dictionary [8] was used to generate AIS08 estimates for all AIS98 codes. Where particular AIS98 codes appeared in the dictionary map on more than one occasion, the first listed AIS08 code was used - generally, this was the default ('NFS') code. An exception was if the AIS08 mapping options differed in their severity levels; in such instances the first listed AIS08 code with the lowest available severity level was used in keeping with conservative AIS coding rules [8]. Where no AIS08 equivalent was provided in the dictionary map, this was noted. Once mapping had been completed, AIS08-based ISS values were calculated from the mapped codes.

In order to examine the potential effect of mapping on injury severities in more detail, AIS98 codes which had been assigned most and least frequently in the VSTR dataset were examined. Of the 1341 codes in the AIS98 dictionary, two subgroups were identified based on code occurrence in the VSTR - codes which had been assigned an average of 100 times per year or more were deemed 'frequently assigned', and those which had been assigned an average of once per year or less were deemed 'rarely assigned'.

Where cerebral SDH codes had been assigned in AIS98, accompanying free text descriptions were searched for text strings such as 'tiny', 'thin' and 'small' within the descriptions, as well as for measurements of thickness and volume (for example, a SDH less than 6 mm thick). Once these had been performed, applicable descriptions were manually reviewed to assess whether detected injury descriptions were relevant. Where the free text description indicated that the most appropriate AIS08 code differed from the AIS08 code in the dictionary map, this was noted.

A paired Wilcoxon signed-rank test was used to compare calculated ISS across the whole population using AIS98 and mapped AIS08 codes. Where categorical data was evaluated, chi-square tests were performed where possible, and standardised residuals were assessed in order to identify specific differences between cells [17]. A *p*-value of 0.05 was taken as indicative of statistical significance, and 95% confidence intervals were calculated using Wilson's asymptotic calculation method [18] where appropriate. To compensate for the number of tests performed, however, a conservative *p*-value of 0.01 was used to indicate significance in statistical comparisons.

## Results

Over the eight year period evaluated, 32134 patients with a total of 128280 coded injuries were included in the VSTR. Using the Victorian major trauma criteria, 15471 (48%) of the patients were classified as major trauma with a total of 85180 coded injuries. Of these patients, 12819 had an ISS >15 and they represented 83% of major trauma patients, or 40% of the total patient group.

Table 1 shows the changes in AIS injury severity (AIS level) when all 1341 codes in the AIS 98 dictionary are mapped forwards from AIS98 to AIS08. Within the dictionary map, 4.5% of codes had decreased in severity, while 0.7% increased; just over 12% of codes either did not currently have an AIS08 map, or had multiple possible AIS08 options of different severities when mapping the AIS98 code. There were differences between these proportions and the corresponding proportions of VSTR-assigned AIS98 codes seen in the same table. In practice, the majority of coded injuries (74.4%) did not change in severity when mapped to AIS08. Only a negligible proportion (0.1%) increased in severity, while almost 13% of the injuries decreased in severity - in other words, the number of injuries which decreased in severity was more than 250 times greater than the number which increased in severity. In addition, 12.8% of injuries either could not be mapped to AIS08 (10.5%), or had some uncertainty as to the accuracy of the severity of the mapped codes (2.3%). These differences were statistically significant (p < 0.001) - those AIS98 codes which increased in severity occurred less frequently in practice, while those which decreased in severity occurred more frequently.

**Table 1:** AIS severity changes for AIS98 dictionary codes and VSTR dataset injuries when mapping to AIS08.

AIS injury severity	AIS98 dictionary codes		VSTR dataset injuries		Overall chi square
		
	Codes	Incidence	Injuries	Incidence	
Same in AIS08	1109*	82.7%	95419	74.4%	*Χ*^2 ^= 188.25; p < 0.001
Increases in AIS08	9 *	0.7%	65	0.1%	
Decreases in AIS08	61 *	4.5%	16318	12.7%	
Multiple mapping options in AIS08 (available options have different severity levels)	9 *	0.7%	2945	2.3%	
No map currently available (no AIS08 map in dictionary map)	153	11.4%	13533	10.5%	

**Total**	**1341**	**100.0%**	**128280**	**100.0%**	

* = p < 0.01 on examination of standardised residuals for *Χ*^2 ^test (|z score| >2.58)

### Differences between rarely and frequently assigned AIS98 codes

Of the 1341 codes in the AIS98 dictionary, only 1180 had been assigned at least once. The incidence of codes assigned showed a highly skewed distribution. There were 43 codes (listed in Table 2) which had been frequently assigned; these represented only 3.2% of the available codes in the AIS98 dictionary but accounted for 44.0% of the injuries sustained. Conversely, more than half of the codes in the AIS98 dictionary (679 codes, or 50.6% of available codes) were rarely assigned; this included 161 codes which had not been assigned at all in the 8-year period (Table 3).

**Table 2:** Frequently assigned AIS98 codes in VSTR, with dataset incidence and effect of mapping to AIS08.

Incidence	AIS98 code	Brief injury description	AIS severity level if mapped to AIS08
2774	**140684.3**	Cerebral subarachnoid haemorrhage	Decreased
2477	**210602.1**	Minor(superficial) skin laceration to face	Same
2152	**650620.2**	Lumbar spine fracture - transverse process	Same
1993	**210402.1**	Skin contusion/haematoma to face	Same
1974	**140652.4**	Small cerebral subdural haematoma	Same
1973	**150200.3**	Base of skull fracture - NFS	Same
1778	**110402.1**	Skin contusion/haematoma to scalp	Same
1737	**752200.2**	Clavicle fracture	Same
1606	**851606.2**	Fibula fracture - head/neck/shaft	Same
1597	**441406.3**	Unilateral lung contusion	Decreased
1586	**110602.1**	Minor(superficial) skin laceration to scalp	Same
1435	**442202.3**	Thoracic cavity injury with haemo-/pneumothorax, NFS	Multiple severities available
1344	**752804.3**	Radius fracture - open/displaced/comminuted	Decreased
1318	**810202.1**	Skin abrasion to lower extremity	Same
1295	**650420.2**	Thoracic spine fracture - transverse process	Same
1291	**250800.2**	Maxilla fracture - NFS	Same
1289	**450220.2**	Rib cage fracture - 2-3 rib fractures any location	Same
1287	**852600.2**	Pelvic fracture - NFS	Same
1277	**650430.2**	Thoracic spine fracture - vertebral body, NFS	Same
1277	**852602.2**	Fractured pelvis - closed/undisplaced	No map currently available
1215	**710202.1**	Skin abrasion to upper extremity	Same
1194	**852604.3**	Pelvic fracture - open/displaced/comminuted	Same
1186	**450232.4**	Rib cage fracture - >3 ribs on one side,≤3 on other side, with haemo-/pneumothorax	No map currently available
1160	**853422.3**	Tibia fracture - shaft, open/displaced/comminuted	Multiple severities available
1087	**851814.3**	Femur fracture - shaft	Same
1085	**450804.2**	Sternum fracture	Same
1070	**753000.2**	Scapula fracture	Same
1049	**160202.2**	Concussive injury - unconsciousness < 1 hr	Same
1028	**710602.1**	Minor(superficial) skin laceration to upper extremity	Same
1026	**650432.2**	Thoracic spine fracture - vertebral body, minor compression (< = 20% loss of anterior height)	Same
1022	**910200.1**	Skin abrasion, multiple regions or NFS	Same
984	**441410.4**	Bilateral lung contusion	Decreased
964	**810402.1**	Skin contusion/haematoma to lower extremity	Same
958	**210202.1**	Skin abrasion to face	Same
948	**752604.3**	Humerus fracture - open/displaced/comminuted	Decreased
929	**251800.2**	Zygoma fracture	Decreased
917	**510402.1**	Skin contusion/haematoma to abdomen	Same
886	**810602.1**	Minor(superficial) skin laceration to lower extremity	Same
883	**753204.3**	Ulna fracture - open/displaced/comminuted	Decreased
870	**450222.3**	Rib cage fracture - 2-3 rib fractures any location, with haemo-/pneumothorax	No map currently available
849	**650230.2**	Cervical spine fracture - vertebral body, NFS	Same
826	**450212.1**	Rib cage fracture - 1 rib	Same
800	**210600.1**	Skin laceration to face, NFS	Same

**Table 3:** Occurrence of rarely assigned AIS98 codes in VSTR.

Occurrences in VSTR dataset	Number of AIS98 codes with this incidence	% of available AIS98 codes (out of 1341 codes)
0	161	12.0%
1	130	9.7%
2	111	8.3%
3	67	5.0%
4	61	4.5%
5	40	3.0%
6	49	3.7%
7	34	2.5%
8	26	1.9%

**Total**	**679**	**50.6%**

Table 4 demonstrates the changes in injury severity when both rarely assigned and frequently assigned VSTR dataset injuries are mapped from AIS98 to AIS08. Six of the nine codes which increased in severity when mapped to AIS08 were rarely or never assigned, while none of the frequently assigned codes increased in severity. While AIS98 codes which decreased in severity in AIS08 accounted for only 2.5% of rarely assigned codes and 4.5% of the overall AIS98 codeset (Table 1), they made up 16.3% of frequently assigned codes.

**Table 4:** AIS severity changes in AIS08 for rarely assigned and frequently assigned AIS98 codes in VSTR.

AIS injury severity	All AIS98 codes	Rarely assigned codes		Frequently assigned codes	
		
		Codes	% of code type	Codes	% of code type
Same in AIS08	1109	581	85.6%	31	72.1%
Increases in AIS08	9	6	0.9%	0	0.0%
Decreases in AIS08	61	17	2.5%	7	16.3%
Has multiple severity levels in AIS08	9	1	0.1%	2	4.7%
No map currently available	153	74	10.9%	3	7.0%

**Total**	**1341**	**679**	**100.0%**	**43**	**100.0%**

### Effect of AIS98 codes missing from the dictionary map

Of the 128280 injuries, 13533 had been assigned an AIS98 code which was not listed in the dictionary map (shown earlier in Table 1). These injuries were spread evenly across the VSTR population, so that 10597 of the 32134 patients in the VSTR (33.0%) had sustained at least one injury which could not be mapped to AIS08. The majority of these patients had sustained other injuries which could be mapped, permitting calculation of at least a partial ISS estimate. However, 1565 patients (4.9% of all study patients) had been assigned AIS98 codes where none of these codes could be mapped to AIS08 and hence could not have any AIS08-based ISS calculated.

Table 5 summarises the injury types sustained by the 1565 patients for whom no codes could be mapped from AIS98 to AIS08. Injuries to the pelvis and its associated bony joints, which underwent extensive classification revision in AIS08, were the most common type of injury which could not be mapped. This was followed by combined thoracic injury codes which had been split into two codes in AIS08. Although the majority of these patients (1451, or 92.7%) only sustained one injury, there were some patients with multiple injures where none could be mapped.

**Table 5:** Injury types sustained by 1565 patients for whom no codes could be mapped to AIS08.

Injury type	Number of injuries	Incidence
Concussive closed head injury	131	7.7%
Other injury to head or face	48	2.8%
Combined thoracic injury code	498	29.3%
Other injury to chest or abdomen	65	3.8%
Upper limb injury	142	8.4%
Pelvic or pelvic joint injury	507	29.8%
Lower limb injury	73	4.3%
Burn injury	235	13.8%

**Total**	**1699**	**100.0%**

### Effect on ISS and major trauma classification

Out of 30569 patients with a calculable ISS using AIS08, 11960 (39.1%) had an ISS which differed from the ISS calculated using AIS98 codes. More than 98% of patients whose ISS changed using AIS08 had a decrease in ISS; only 222 patients (0.7% of patients with a calculable ISS) had a higher ISS using mapped AIS08. Overall, the median ISS decreased between AIS98 (10; interquartile range 8-20) and mapped AIS08 (9; interquartile range 5-17). Paired Wilcoxon signed rank testing demonstrated that overall ISS was significantly lower using mapped AIS08 (p < 0.001).

Table 6 demonstrates the effect of mapping from AIS98 to AIS08 on calculated ISS by grouping. When codes from all 32134 patients were evaluated, the number of patients with a calculated ISS >15 decreased by 28.1% using mapped AIS08 data, compared with AIS98 data. For 3649 patients, the ISS dropped from above to below ISS 15, while for 44 patients, the ISS increased from less than to greater than 15. The decrease in ISS >15 patients was also assessed for the subset of 21537 patients whose AIS98 codes could all be mapped to AIS08. For this group, the decrease in the number of ISS >15 patients was smaller, with only an 18.2% decrease in AIS08.

**Table 6:** Change in ISS level (ISS <15 or ISS >15) when using AIS dictionary map.

	All patients		Only patients with all codes mappable to AIS08	
	
	Patients	Incidence	Patients	Incidence
**ISS >15 using AIS98**				
ISS >15 using mapped AIS08	8894	27.7%	6125	28.4%
ISS <15 using mapped AIS08	3649	11.4%	1406	6.5%
No injuries in current AIS map	276	0.9%		
**ISS <15 using AIS98**				
ISS >15 using mapped AIS08	44	0.1%	37	0.2%
ISS <15 using mapped AIS08	17982	56.0%	13969	64.9%
No injuries in current AIS map	1289	4.0%		

**Total patients**	**32134**	**100.0%**	**21537**	**100.0%**

Total ISS >15 patients using AIS98	12819		7531	
Projected decrease using AIS08 *(95% CI)*	3605	28.1% *(27.4-28.9%)*	1369	18.2% *(17.3-19.1%)*

The type of Victorian major trauma criteria which was met was assessed for all patients (Table 7). A total of 9267 patients met at least one of the major trauma criteria other than ISS >15 (death after injury, ventilated ICU stay or urgent surgery). Use of these criteria lessened the decrease in the number of patients classified as major trauma when data was mapped to AIS08. If ISS >15 had been the sole major trauma criterion, the number of major trauma patients would have decreased by up to 30.3%. However, using both ISS >15 and the additional Victorian criteria, the decrease was only 17.3%.

**Table 7:** Victorian major trauma criteria met using AIS98 and (mapped) AIS08.

	Criteria met using AIS98		(Projected) criteria met using AIS08	
	
	Patients	Incidence	Patients	Incidence
ISS >15 only	6204	40.1%	3528	27.5%
Both ISS >15 and other criteria	6615	42.8%	5410	42.3%
Non-ISS criteria only	2652	17.1%	3857	30.1%

**Total major trauma patients**	**15471**	**100.0%**	**12795**	**100.0%**
Projected decrease using AIS08 *(95% CI)*	17.3% *(16.7-17.9%)*			

**Total ISS >15 patients**	**12819**	**82.3%**	**8938**	**69.9%**
Projected decrease using AIS08 *(95% CI)*	30.3% (29.5-31.1%)*			

* = Estimate includes 276 patients for whom an ISS could not be calculated in AIS08

### Evaluation of free text descriptions for cerebral SDH

A total of 3541 cerebral SDH injuries were identified; overall, 11.0% of patients in the VSTR had sustained a cerebral SDH. The majority of these injuries (2580, or 72.9%) had an AIS98 code of level 4 severity assigned, and the remaining 961 patients had a level 5 severity. When using the dictionary map only, 329 of the AIS level 5 injuries decreased to level 4, and all 606 NFS codes decreased in severity from level 4 to level 3 (Table 8). When free text descriptions were evaluated, the mapped AIS08 codes for 319 injuries (accounting for 9.0% of cerebral SDH injuries) changed; these changes affected 37 of the NFS codes, 251 of the small (unilateral) codes and 31 of the (small) bilateral codes. All but ten of these 319 changes involved a change in the severity of the AIS08 code from what had originally been mapped. A total of 251 SDH codes changed to a lower severity than had originally been mapped in AIS08 while 58 codes changed to a higher severity. This included 31 bilateral SDH injuries which remained at level 5 severity following mapping to AIS08 after the thickness of the haematoma on one side was noted to be >10 mm. It was also noted that 37 (6.1%) of the NFS codes contained information in the free-text descriptors which permitted mapping to a more specific SDH code - so it could be argued that the NFS code should not have been assigned in AIS98.

**Table 8:** Conversion of 3541 cerebral SDH codes from AIS98 to AIS08 using two mapping techniques.

AIS08 code AIS98 code	140650.3 NFS	140651.3 Tiny (< 6 mm)	140652.4 Small (6-10 mm)	140654.4 Small bilateral	140655.5 Large (> 10 mm)	140656.5 Large bilateral
140650.4 NFS	**606** *569*	**-** *10*	**-** *27*	**-** *-*	**-** *-*	**-** *-*
140652.4 Small (≤1 cm)	**-** *-*	**-** *251*	**1974** *1723*	**-** *-*	**-** *-*	**-** *-*
140654.5 Small bilateral	**-** *-*	**-** *-*	**-** *-*	**329** *298*	**-** *31*	**-** *-*
140656.5 Large (> 1 cm)	**-** *-*	**-** *-*	**-** *-*	**-** *-*	**-** *-*	**632** *632*

**Total at severity level**	**AIS08 level 3**		**AIS08 level 4**		**AIS08 level 5**	
	**606** *830*		**2303** *2048*		**632** *663*	

Results of mapping from AIS98 using the dictionary map are shown in bold; results using the dictionary map augmented by free text evaluation are shown in italics. For each AIS98 SDH code (rows), the number of AIS08 codes derived are shown.

## Discussion

This is the largest study evaluating the effect of AIS mapping performed to date. It confirmed that using the dictionary map to convert a trauma dataset from AIS98 to AIS08 significantly decreases the overall injury severity of the dataset. This has a substantial effect on the number of patients classified as major trauma, although in the VSTR, the decrease in major trauma numbers was offset by the use of major trauma criteria other than just an ISS >15. The inclusion of non-major trauma patients did not result in a substantial number of 'new' patients being classified as major trauma using AIS08, as very few patients recorded an increase in ISS in AIS08. This indicates that estimates from previous studies which have only used major trauma patients in evaluating changes in major trauma classification are likely to have been reasonably accurate for the populations which were evaluated.

The current study also illustrated that calculating the change in the number of classified major trauma patients may depend on whether AIS-based criteria (such as ISS >15) alone are used. The number of major trauma patients decreased by 17.3% using Victorian major trauma criteria, but decreased by 30.3% when using an ISS >15 alone. These findings are comparable with previous studies [9-11] which showed that between 14 and 24% of patients currently designated as major trauma using an ISS >15 would be reclassified as non-major in AIS05/08, although some variation in these percentages is expected due to the different trauma datasets or populations used.

### Effect of AIS98 codes missing from the dictionary map

The absence of 153 AIS98 codes from the dictionary map represents a second factor which has a substantial impact on estimates of major trauma classification using AIS08. These codes disproportionately affect the patient population, as one third of patients had at least one of these codes (potentially affecting ISS), and a small but not insignificant proportion (4.9%) of patients could not have any ISS calculation performed. The injuries sustained by these patients were heavily biased towards two particular injury types (pelvic injuries and complex thoracic injuries). Conversely, it may be said that the set of AIS98 codes which can be mapped to AIS08 is biased away from these injury types.

The proportion of injury codes which experienced mapping difficulties overall is also worth comment. A total of 16383 injuries were identified as changing in injury severity (increasing or decreasing) when mapped to AIS08. However, there were 16478 injuries which either could not be mapped to an AIS08 injury, or mapped to a known severity level. In other words, the estimate of the change in severity of the study population contains a margin of uncertainty which is greater than the measurable changes from AIS98 to AIS08. Consequently, the issue of missing codes carries major implications for the conversion of existing AIS98-coded trauma datasets to AIS08, as they introduce an unacceptably large degree of uncertainty into the resultant (mapped) codeset. This forms a considerable barrier to the ideal aim of accurate and complete mapping of existing data to AIS08, which is necessary to perform meaningful, unbiased comparisons within a population over time, or between trauma systems using different AIS versions.

### Use of free text descriptions in AIS mapping

Evaluation of free text descriptions for cerebral SDH data showed that this information improved the accuracy of mapping to AIS08. Compared with the codes automatically assigned by the dictionary map, the AIS08 codes for a small but substantial proportion of SDH injuries changed when free text information was used. In the vast majority of these patients, the severity of the mapped AIS08 code also changed. It is unlikely that the percentages of altered codes reported in this study would remain the same across other injury types. However, this validates the use of free text evaluation in determining codes which may have been erroneously assigned in AIS98 (for example, the 'NFS' codes which should have been coded as 'small'), or in assigning AIS08 maps which are more accurate than may be provided by the dictionary map alone.

### Limitations

The VSTR dataset encompasses a complete population-based trauma system which is not subject to many of the reporting and selection biases inherent in many regional and national-level registries [19]. However, the incidence and distribution of injuries within the VSTR may not be the same as that in other regions or countries. For example, the occurrence of penetrating trauma in the US is known to be higher than in Australia, hence US trauma datasets are likely to include more injuries to deeper torso, neck and brain structures. This will affect the proportions of the injury codeset which increase or decrease in severity, or which cannot be coded. In turn, this will affect both the proportion of patients classified as major trauma patients, and the accuracy of estimates made using mapping.

A second limitation of this study is that the measured effect of AIS08 adoption on major trauma applies only to the currently used major trauma criteria. Most of the VSTR patients who are classified as major trauma met an ISS threshold which was established in 1987 [13], based on management and survival expectations which are now a generation old. A more recently developed guideline has suggested NISS >15 as an inclusion criterion for severely injured patients [20], but for current AIS versions there is no published evidence to support any particular ISS or NISS threshold. As ISS or NISS thresholds form part or all of the inclusion criteria for many trauma registries, the adoption of AIS05 or AIS08 should coincide with a re-evaluation of the appropriateness of the thresholds being used to denote major trauma in both the VSTS and in other trauma registries.

It should also be noted that the present study is only the second to use the AIS dictionary map, and used an identical mapping technique to the other study which has used the map [9]. No other guidelines for the use of this map have been released by either the AIS' developers or by other researchers. We believe that the method used to assign AIS08 maps was logical; that it adhered to the rules of AIS coding set out by the AIS' developers [4,7,8]; and that it demonstrated the simplicity which would be required to automatically map large datasets using a computer algorithm. It could be argued that part of the reason for the observed decrease in ISS in some patients was due to the mapping technique used. However, Table 1 shows that only 2.3% of VSTR injuries had been assigned AIS98 codes which had differing severity levels available in AIS08. At worst, therefore, the mapping technique used accounts for only a small proportion of the observed decrease in severity using AIS08.

### Future directions

It is likely that many (if not all) of the 153 AIS98 codes for which no AIS08 equivalent has been assigned in the AIS dictionary map may have valid equivalents in practice, including combined thoracic injury codes where AIS08 codes for the individual injury components exist, and closed or undisplaced fractures of upper limb long bones. There are also instances where specificity has been removed (such as burn injuries with hand, face or perineal involvement) or where the anatomical nomenclature has changed (such as pelvic fractures).

It should therefore be possible to determine plausible AIS08 equivalents for many of these currently 'unmappable' AIS98 codes, without affecting the dictionary map already in place within the AIS08 dictionary. In other words, any enhancement to the existing map must complement, rather than replace it. The goal of an 'enhanced map' would be to improve the proportion of injuries for which the AIS98 to AIS08 map gives the same AIS08 code (or at least the same AIS08 severity level) as if the patients' injuries had been directly coded using AIS08. It is possible that removing some or all of the 'unknown quantity' from an AIS mapping exercise such as the current study would improve comparability to the point where the results across a given trauma population were of acceptable quality.

Similarly, the aim in using free text descriptions is to provide further improvements in the accuracy of an AIS08-based estimate from AIS98-coded data. Evaluation of cerebral SDH codes showed that the accuracy of data conversion can be enhanced by using this method, and it may be a technique which can be employed without directly affecting the current dictionary map - again, this method would complement, rather than replace, the current mapping tool. This technique of mapping is not new: it has also been used in ICD coding to set precedents for the use of free text information in migrating data between versions of a codeset, a practice known as 'interactive conversion' [21]. While the use of free text descriptions may seem labour-intensive, it is worth noting that if an existing registry commences using AIS08, earlier AIS98-coded data need only be converted to AIS08 once; moreover, it would be far quicker and cheaper than re-coding using the original medical records of each patient in a registry.

## Conclusions

Compared with AIS98, adoption of AIS08 leads to a decrease in the overall injury severity of the trauma population. Depending on the assumptions made, the number of patients classified as major trauma is estimated to decrease by between 17.3% and 30.3%. These estimates may vary considerably due to differing major trauma criteria, and are also affected by the absence of a substantial number of AIS98 codes from the dictionary map. These absences affected AIS98 to AIS08 mapping, and potentially the accuracy of the ISS calculation, in 33.0% of study patients and prevented the calculation of any ISS value in 4.9% of patients.

Relying solely on the map provided in the AIS08 dictionary does not provide AIS08 estimates or derived ISS estimates which can be reliably used in practice. However, it may be possible to derive plausible AIS08 equivalents for many of the 153 AIS98 codes currently lacking maps. Where available, using free text descriptions may improve the accuracy of mapped AIS data.

## Conflict of interests statement

There are no conflicts of interest or external funding sources to acknowledge in the preparation of this paper.

## Authors' contributions

CSP conceived the study, performed all data analysis, and created the first draft of the manuscript. Both authors revised the manuscript, developed ideas for additional analysis and discussion, edited and gave final approval to the manuscript.

## References

[B1] Committee on Medical Aspects of Automotive Safety Rating the severity of tissue damage. I. The Abbreviated Scale JAMA 1971 215 277 280 10.1001/jama.215.2.277 5107365

[B2] Baker SP O'Neill B Haddon W Long WB The injury severity score: a method for describing patients with multiple injuries and evaluating emergency care J Trauma 1974 14 187 196 10.1097/00005373-197403000-00001 4814394

[B3] Osler T Baker SP Long W A modification of the injury severity score that both improves accuracy and simplifies scoring J Trauma 1997 43 922 925 10.1097/00005373-199712000-00009 9420106

[B4] Association for the Advancement of Automotive Medicine The Abbreviated Injury Scale 1990 Revision - Update 98 1998 Barrington, IL: Association for the Advancement of Automotive Medicine PMC3256842 22105401

[B5] Gennarelli TA Wodzin E AIS 2005: A contemporary injury scale Injury 2006 37 1083 1091 10.1016/j.injury.2006.07.009 17092503

[B6] Skaga NO Eken T Hestnes M Jones JM Steen PA Scoring of anatomic injury after trauma: AIS 98 versus AIS 90 - do the changes affect overall severity assessment? Injury 2007 38 84 90 10.1016/j.injury.2006.04.123 16872609

[B7] Gennarelli TA Wodzin E (Eds) The Abbreviated Injury Scale 2005 2005 Barrington, IL: Association for the Advancement of Automotive Medicine

[B8] Gennarelli TA Wodzin E (Eds) The Abbreviated Injury Scale 2005 - Update 2008 2008 Barrington, IL: Association for the Advancement of Automotive Medicine

[B9] Palmer CS Niggemeyer LN Charman D Double coding and mapping using Abbreviated Injury Scale 1998 and 2005: Identifying issues for trauma data Injury 2010 41 948 954 10.1016/j.injury.2009.12.016 20074729

[B10] Royal Perth Hospital Royal Perth Hospital Trauma Services Report - 2007 Perth 2008 http://www.rph.wa.gov.au/pdf/Trauma_Report_2007.pdf

[B11] Salottolo K Settell A Uribe P Akin S Slone DS O'Neal E Mains C Bar-Or D The impact of the AIS 2005 revision on injury severity scores and clinical outcome measures Injury 2009 40 999 1003 10.1016/j.injury.2009.05.013 19524239

[B12] Wang D Lu L Assessment of the injury severity score in evaluation of multiple maxillofacial injuries Chin J Stomatol 2008 43 646 649 19087635

[B13] Boyd CR Tolson MA Copes WS Evaluating trauma care: the TRISS method. Trauma Score and the Injury Severity Score J Trauma 1987 27 370 378 10.1097/00005373-198704000-00005 3106646

[B14] Barnes J Hassan A Cuerden R Cookson R Kohlhofer J Comparison of injury severity between AIS 2005 and AIS 1990 in a large injury database Ann Proc Assoc Adv Automot Med 2009 53 83 89 PMC3256797 20184835

[B15] Copes WS Lawnick M Champion HR Sacco WJ A comparison of Abbreviated Injury Scale 1980 and 1985 versions J Trauma 1988 28 78 86 10.1097/00005373-198801000-00011 3339666

[B16] Victorian State Trauma Registry 2007-08: summary report Victorian Government Department of Human Services 2009 http://www.health.vic.gov.au/trauma/publications/0708vstorm-sumrep.pdf retrieved 7 September 2010

[B17] Sheskin D Handbook of parametric and nonparametric statistical procedures 2004 3 New York: Chapman & Hall/CRC

[B18] Brown L Cai T DasGupta A Confidence intervals for a binomial proportion and asymptotic expansions Ann Stat 2002 30 160 201 10.1214/aos/1015362189

[B19] Cameron PA Gabbe BJ Cooper DJ Walker T Judson R McNeil J A statewide system of trauma care in Victoria: effect on patient survival Med J Aust 2008 189 546 550 19012550 10.5694/j.1326-5377.2008.tb02176.x

[B20] Ringdal KG Coats TJ Lefering R Di Bartolomeo S Steen PA Røise O Handolin L Lossius HM The Utstein template for uniform reporting of data following major trauma: A joint revision by SCANTEM, TARN, DGU-TR and RITG Scand J Trauma Resusc Emerg Med 2008 16 7 10.1186/1757-7241-16-7 18957069 PMC2568949

[B21] Shulz S Zaiss A Brunner R Spinner D Klar R Conversion problems concerning automated mapping from ICD-10 to ICD-9 Methods Inf Med 1998 37 254 259 9787625

